# A Review on Bast-Fibre-Reinforced Hybrid Composites and Their Applications

**DOI:** 10.3390/polym15163414

**Published:** 2023-08-15

**Authors:** Teboho Clement Mokhena, Asanda Mtibe, Thabang Hendrica Mokhothu, Mokgaotsa Jonas Mochane, Maya Jacob John

**Affiliations:** 1 DSI/Mintek-Nanotechnology Innovation Centre, Advanced Materials, Mintek, Randburg 2125, South Africa; 2 Centre for Nanostructures and Advanced Materials, Chemicals Cluster, CSIR, Pretoria 0001, South Africa; amtibe@csir.co.za (A.M.); mjohn@csir.co.za (M.J.J.); 3 Department of Chemistry, Durban University of Technology, Durban 4000, South Africa; thabangm1@dut.ac.za; 4 Department of Life Sciences, Central University of Technology Free State, Bloemfontein 9301, South Africa; mochane.jonas@gmail.com; 5 Department of Chemistry, Nelson Mandela University, Port Elizabeth 6001, South Africa

**Keywords:** bast fibres, hybrid composites, mechanical properties, thermal properties, flame retardant properties

## Abstract

The development of eco-friendly products to protect the environment has become a topical subject in the research and industrial communities. This is a result of strict environmental regulations necessitating the development of novel strategies to reduce our reliance on petroleum-based products, which exert a negative effect on our ecosystem. Bast-fibre-based hybrids have been extensively studied for various applications due to their eco-friendliness and cost effectiveness. There is a very limited number of review articles covering the properties and preparation of bast-fibre-based hybrid composites. This review is designed to provide an overview of the preparation and application of bast-fibre-based hybrid composites. It covers the thermal properties, mechanical properties, moisture absorption and flame-retardant properties of bast hybrid composites. This review not only summarises recent advances on the use and preparation of bast hybrid composites, it also presents a future outlook.

## 1. Introduction

Renewable materials are a new class of materials with huge potential to replace traditional petroleum-based materials in some applications [1,2,3]. Natural fibres derived from natural renewable materials, i.e., animals and plants, are described as new ‘green’ reinforcing materials, and can be used to improve the overall properties of various polymers [4]. This is as a result of their attractive attributes, such as their light weight, availability, inexpensiveness, high specific mechanical properties, biocompatibility, biodegradability, and non-toxicity. It has been recognised that the inherited hydrophilicity of natural fibres limits their broad application, especially when considering long-term durability under cyclic loading [1]. Some reports have demonstrated that the presence of natural fibres in composite materials can adversely affect mechanical performance [1], emphasising that the significant moisture adsorption caused by the fibres’ presence leads to a decrease in tensile modulus and strength [1]. Given the limitations of natural fibres, research has been dedicated in mitigating these drawbacks at relatively low cost.

Over the span of decades, increasing attention has been given to combining two fibres from different sources to overcome these hurdles, in order for these materials to be able to compete with synthetic-based fibres at the commercialisation stage [5]. With glass fibres dominating the reinforced composites, *viz*., 95% volume, the combination of glass fibres with natural fibres (coir [6], bamboo [7], jute [8,9], areca core sheath [8], and flax [10]) has attracted attention from researchers and industry from both economic and ecological perspectives [6,7,11]. Glass fibre is utilised as a reinforcing agent due to the properties of the resulting materials, which include excellent mechanical properties, non-biodegradability, moisture insensitivity, corrosion resistance, and relatively low cost. Nonetheless, other synthetic fibres have been combined with natural fibres to develop new materials with fascinating properties for advanced applications when compared to individually reinforced polymers. Therefore, the hybridisation of fibres, e.g., synthetic, natural fibres, or other fibres, to serve as reinforcement for various polymers offers a new platform to explore with the aim of reaping the benefits of both. In the literature, bast fibres have received more attention due to their promising performance when compared to synthetic fibres, e.g., glass fibres, as well as other natural fibres [2]. The driving force has been their attractive attributes, which include renewability, light weight, abundant availability, and excellent mechanical performance.

Bast fibres are one of the plant fibres commonly used in reinforced polymer composites. They are long, and are obtained from the exterior part of the plant stalk. These fibres are often used in the automotive industry to replace traditional fibres in car components, such as in door panels, seatbacks, etc. Bast fibres exhibit drawbacks similar to those of other natural fibres, including excessive moisture absorption, poor thermal stability, inherent variability, and high flammability. Hybridisation with other fibres has been the most promising solution for overcoming these limitations. Synthetic fibres (e.g., glass fibres, carbon fibres, and basalt fibres) and other natural fibres have been hybridised with bast fibres to develop hybrid composite materials that not only address the limitations of bast fibres, but also with the aim of developing complete composite materials with balanced overall properties [12]. Most published reviews have been based on single bast-fibre-reinforced composites [13,14,15,16,17]. To the best of our knowledge, there is a limited number of review papers dedicated to bast-fibre-based hybrid composites [18]. This review’s primary objective is to delve into the literature on bast hybrid composite preparation. The thermal, mechanical, moisture absorption, and flame resistance properties of the resultant hybrid composites are also discussed. We conclude with recent challenges and trends with regard to overcoming the issues associated with these materials and their application in different fields.

### Bast Fibre Properties and Economic Benefits

Bast fibres can be obtain all around the world, and grow very quickly when compared to traditional natural fibres, e.g., wood. Thus, these fibres have been employed since ancient times, mostly as a replacement for wood. Trade for bast fibres has increased rapidly over the years. India is the highest exporter, followed by Tanzania (see Table 1) (https://wits.worldbank.org/trade/comtrade/en/country/ALL/year/2021/tradeflow/Exports/partner/WLD/product/530310, accessed on the 9 September 2022). The global export increased by ~27% from USD 39 million in 2017 to USD 48 million in 2019. This indicates the interest in using these fibres across multiple sectors. The recent activity in different sectors regarding the modification of bast fibres to overcome their moisture absorption in order to broaden their application in transportation and sport equipment indicates that bast fibres play a critical role in empowering and alleviating poverty, especially for farmers from developing countries (e.g., Tanzania, India, etc.) (https://renewable-carbon.eu/news/native-bast-fibres-for-buses-boards-and-automotive-equipment/, accessed on the 12 September 2021). The light weight and mechanical performance of bast fibres have been the most important properties considered regarding their application in various sectors. Despite these fibres mostly being used in textile applications, the current trend has seen their transition to being one of the best reinforcing agents among polymer composites.

Natural-fibre-reinforced composites have attracted attention because of their fascinating properties, which include affordability, renewability, biodegradability, and excellent specific mechanical properties. Table 2 summarises the characteristics of bast fibres when compared to other natural and synthetic fibres [12,19,20,21,22]. It can be seen that these fibres are characterised by their high specific mechanical properties, which can be correlated with the fibril angle [19]. The four most frequently exploited bast fibres are jute, kenaf, flax, hemp, and ramie. Other bast fibres such as Roselle also fall under the category of bast fibres. These fibres are grown commercially in different continents (Asia, Europe, Africa, North and South America) and are often employed in fabric materials, papers, and cement products, as well as in green reinforcing agents for various polymeric materials. This is due to their fascinating features, such as inexpensiveness, light weight, biodegradability, renewability, excellent tensile strength and modulus.

## 2. Chemical Treatment of Fibres

Similar to any other natural plant fibre, bast fibres are inherently hydrophilic because of their constituents, *viz.,* cellulose, hemicellulose and lignin [3,21,22,23,24,25,26,27,28]. These constituents have a large number of hydroxyl groups, which are responsible for the hydrophilic nature of these fibres, and hence their high moisture sensitivity. Despite the fact that high moisture sensitivity is the primary limiting factor for industrial application of plant-based fibres, these surface functional groups are crucial for the chemical modification of the fibres. These modifications improve the polarity of the fibres, which significantly improves interfacial bonding with hydrophobic polymers [20]. Chemical treatment (e.g., alkali treatment, silane treatment) is the process most frequently used to improve the hydrophobicity of fibres. In most cases, alkali treatment is used prior to other treatments in order to remove residues on the surface of the fibres, thus promoting fibrillation and surface roughness [3,28].

### 2.1. Alkali Treatment

The most commonly used treatment for plant fibres is alkali treatment, which is also known as mercerisation [20,21,22]. This process involves the removal of impurities on the fibre surface, thus affording rough surfaces. A sodium hydroxide solution with concentrations ranging between 5 and 20% is usually used for treating plant-based fibres. The time often ranges between 1 and 5 h, depending on the temperature used. Elsewhere, ramie fibres have been treated with 5% NaOH solution for 4 h at room temperature [28]. The obtained treated fibres were washed and neutralised using deionised water containing acetic acid to ensure the complete removal of NaOH. The fibres were then dried in a vacuum oven at 98 °C for hybrid preparation. Fibrillation of the fibres was achieved through partial removal of the matrix that causes the fibrils to adhere together, i.e., gums and pectin. Saw et al. [3] treated jute fibres with 5% NaOH at 30 °C and kept the liquor ratio at 15:1 for 2 h before combining with bagasse fibres to produce hybrid composites. The cracks or pits observed on the surface of the fibres were due to the partial removal of fatty substances and wax.

### 2.2. Coupling Agents

**Figure 1:**
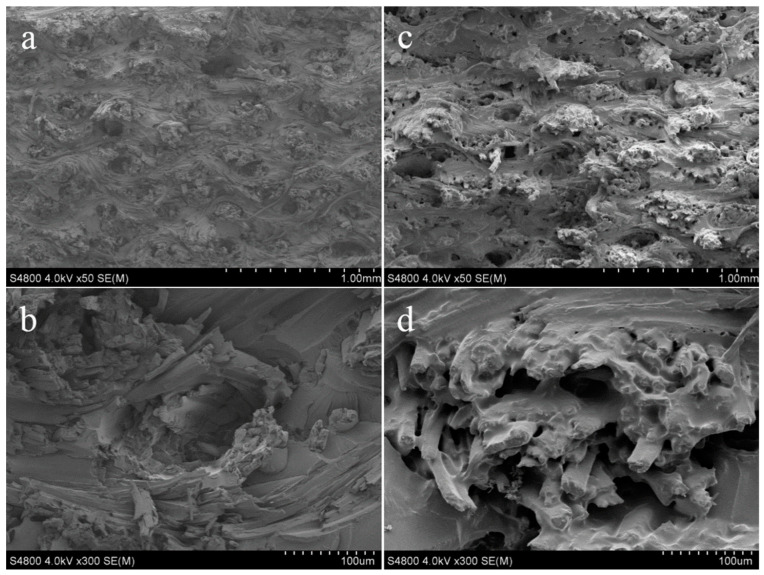
SEM images of tensile fractured for (**a**,**b**) untreated ramie and (**c**,**d**) silane treated ramie fibres hybrid composites. Reproduced from [23].

Silane treatment of ramie fibres was carried out in order to improve the interaction between the fibres and the host polymeric material [23,24,28]. Functionalised ramie fibres were found to be trapped within the host matrix, indicating that there was good wettability between the filler and the matrix (Figure 1c,d) [23]. Due to the strong interaction between the fibres and matrix, there was better stress transfer from the polymer to the fibres, resulting in enhanced mechanical properties. The weak interfacial adhesion between the untreated ramie fibres and the matrix resulted in fibre pullouts, leaving behind holes (Figure 1a,b) [23]. Elsewhere, NaOH treatment for kenaf and pineapple leaf fibre (PALF) was carried out prior to the silane treatment of the fibres [28]. Hybridised and unhybridised composites showed fibre–matrix debonding, fibre pullouts, and breakage, indicating that a large amount of load was being carried by the fibres. Moisture absorption and mechanical properties were found to be dependent on the type of fibre. For instance, PALF with high cellulose content had excellent mechanical capabilities and high moisture absorption, but kenaf fibres caused a significant decrease in both of those properties.

Yet another coupling agent, maleic anhydride grafted polypropylene (MA-g-PP), was found to enhance fibre–polymer interaction [25,27]. This type of modification has also been used together with alkali treatment [27]. Maleic anhydride PP was used by El-sabbagh et al. [26] as a coupling agent for PP to increase the interfacial adhesion between PP and fibres. They found that this enhanced the interfacial adhesion, mechanical properties, and flame resistance performance.

The secondary modification of silane-treated fibres can drastically compromise the interaction between the fibres and the host polymeric material [24]. Li et al. [24] reported that the modification of ramie fibres with ammonium polyphosphate (APP) to impart flame-retardant properties negatively affected the resulting properties. The fibres were treated with 1% silane solution, followed by drying before soaking in 5% APP solution. It was noted that the presence of APP improved the flame-retardant properties, but there were voids between the fibres and the polymer, with clean surface fibres pulled out.

### 2.3. Microbiological Treatment

Biological treatments are recognised for their eco-friendly and energy-saving characteristics [29]. Angelini et al. [29] compared the defibrillation of the ramie fibres using enzymes and NaOH. They used two strains of Clostridium felsineum L (NCIMB 10690 (MIC 10690) and NCIMB 9539 (MIC 9539)) because of their high pectonolytic efficacy. In the case of alkali treatment, the contained fibres were subjected to 2% NaOH and immersed in boiling water for 2 h. The chemical treatment was found to be more efficient at removing hemicellulose and lignin when compared to the two enzymatic strains. However, there was no significant difference between the strains when it comes to the removal of lignin and hemicellulose. In addition, there was no significant difference in elongation at break, tensile strength, or modulus between the fibres obtained using either alkali or enzymatic treatment.

## 3. Bast Hybrid Composites

**Figure 2:**
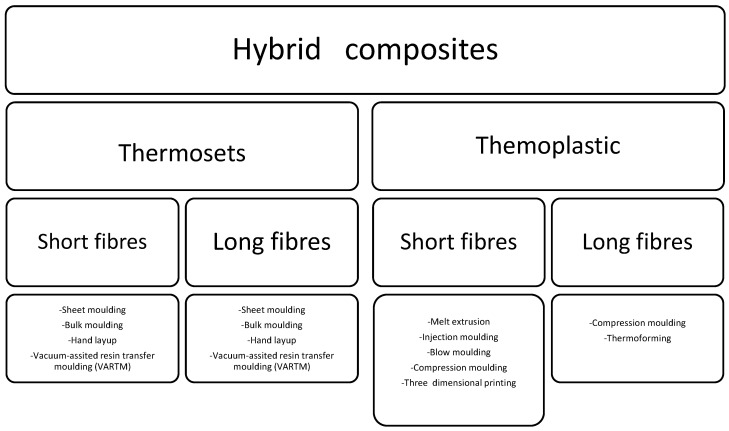
Processing techniques of hybrid composites.

There are several factors, such as fibre loading, the effect of the treatment applied to the fibres, and the stacking of the fibres, that play an important role in the resultant properties of the bast hybrid composites [30,31]. Since these factors are essential for their intended applications, most studies based on bast hybrid composites have been dedicated to finding the balance between these factors [32,33,34,35]. In addition, there have been some studies dedicated to finding a good combination between the fibres, since they display different properties [33,35]. In general, the combination of synthetic and bast fibre results in a composite product with enhanced mechanical performance and low moisture absorption. Synthetic fibres that are often combined with bast fibres include carbon, Kevlar, glass and basalt. The combination of bast as a reinforcing agent with other natural fibres with high impact resistance results in hybrids with inferior attributes compared to single-bast-fibre-reinforced composites [33]. In some studies, the presence of these fillers has been shown to result in a balance of the two fibres in terms of the resulting properties [35]. This indicates that there is an optimal content and/or stacking for attaining the anticipated properties [35]. In addition, the treatment or chemical modification is often required in order to improve interfacial adhesion, which translates to other properties, especially mechanical performance. Additionally, the optimisation of parameters in the manufacturing process plays a significant role in attaining the optimum results with bast hybrid composites. In this respect, the processing technique is directly dependent on the host matrix used, and the fibre structure/shape, and hence their intended applications, as shown in Figure 2 (Table 3) [33,34,35,36,37,38,39,40,41,42,43,44,45,46,47,48,49]. For instance, Indar Reddy and co-workers [34] studied the effect of the hybridisation of two matrices, i.e., polyester and epoxy resins, using a hand layup processing technique. The ratio of jute, glass and pineapple leaf was kept at 1:1:1. It was found that the epoxy-based hybrid of glass/jute and pineapple leaf exhibited superior mechanical perfomance when compared to polyester-based hybrid composites. It was suggested that these composites could be employed in automobiles, electronic casings, and construction.

### 3.1. Thermoplastics

Melt compounding is commonly employed for thermoplastic polymers, since they can be melted and moulded into the desired shape/structure [35,36,37,38,39,40]. These techniques include melt compression, extrusion method, and injection moulding. Some of these techniques can be used alone or together in order to produce the desired hybrid composite products [35]. In addition, melt extrusion and injection moulding have been employed for short fibres, whereas melt compression can be applied for both short and long fibres, as well as woven or non-woven fibres [37]. In the case of compression moulding, higher contents (30–50 wt.%) of fibres can be used to reinforced thermoplastic polymers [38]. The use of more than 30% of fibres within hybrid composites for other melting processing techniques has been reported to reduce the overall performance of the hybrid composites [41,42]. Different thermoplastic polymers, such as polycaprolactone (PCL), polylactic acid (PLA), polypropylene, and polyethylene, have been employed for the preparation of bast hybrid composites [40,41,42]. Hybrid composites composed of a 50:50 ratio of PLA and jute and hemp was prepared using compression moulding by Sahayaraj et al. [40]. The hybridisation resulted in strong interfacial adhesion, leading to improved mechanical performance compared to single-fibre-reinforced composites. In addition, hybridisation using compression moulding can be achieved by stacking layers of fibres. For example, Khoshnava et al. [39] manufactured hybrid composites for construction purposes using compression moulding. Woven kenaf fibres, used as reinforcing filler because of their excellent tensile strength, were introduced, with oil palm empty fruit bunches (EFB) with superior toughness, into polyhydroxybutyrate (PHB) to develop a hybrid composite. Prior to the fabrication of the composite, caustic and silane treatments were used to reduce the fibres’ hydrophilicity and to increase the compatibility of the hybrid filler with the host polymeric components. The samples were arranged in different layouts and layers to achieve the desired composite product. It was noted that the composite made of 11 layers had the potential to replace wood in construction.

### 3.2. Thermosets

The thermoset resins most often used for manufacturing hybrid composites are epoxies [45,46,47,48,49], melamines, phenolics [3], polyesters [30,31], and polyurethanes. Among the thermoset group, epoxy is the most commonly employed for the fabrication of hybrid composite materials [45,46,47,48,49]. In this context, the resin undergoes crosslinking into a three-dimensional network through a curing process to afford a product that cannot be melted or reshaped again. Hybrid composites based on thermosets are often manufactured using vacuum fusion [48,49], compression moulding, and hand lay-up processes [45,46,47]. The latter is often used together with compression moulding in the desired mould [45]. In most cases, long fibres in either woven or non-woven form are preferentially utilised to prepare these hybrid composites because they can be used for structural applications, e.g., civil infrastructure applications. The arrangement of the layers or patterns affords different properties. For instance, Jawaid et al. [45] studied the influence of woven jute fibres on the mechanical performance of tri-layered oil palm empty fruit bunches (EFB). The hybrid composite’s overall mechanical performance was enhanced by the presence of woven jute fibres. When jute was used as stacked on both sides with EFB as the core, the mechanical performance was significantly improved when compared to when jute was used as the core in a tri-layer setting. The improvement of the resultant mechanical characteristics was found to be significantly influenced by the strong interfacial adhesion between the host polymeric material and the jute/EFB/jute fibres.

**Summary**: Other process, such as the solvent evaporation method, have not been utilised to prepare hybrid composites. Regardless of whether long or short fibres are utilised as reinforcing agents in hybrid manufacturing, pretreatment is crucial to the resultant properties. The preparation methods are often chosen with the aim of achieving the desired properties at a relatively low cost. The sensitivity of the natural fibres to long exposure to high heat limits the number of polymers that can be used for the preparation of thermoplastic-based hybrid composites via melt compounding techniques. The limitations of thermosets include recyclability, due to the three-dimensional network formed due to the curing process.

## 4. Effects of Moisture Absorption

Moisture absorption by natural-fibre-based polymer composites (NFBPC) is one of the most common concerns or drawbacks affecting the mechanical and physical properties of the composite for high-end or outdoor applications. The hydrophilic nature of natural fibres causes weakness in the interfacial adhesion between the fibres and the polymer matrix in the composite. The adsorption of moisture by the composites relies on various factors, which include the humidity and temperature, the fibre type, the matrix, the reaction between the matrix and water, the volume fraction of the fibre, voids, and the difference in the water distribution within the composites [50,51,52,53,54,55,56]. Moisture absorption by NFBPC is governed by three different mechanisms of moisture diffusion. The first is the diffusion of water molecules into the microscopic spaces between polymer chains, the second is the diffusion of water molecules via capillary transport into the gaps and flaws at the interface between the polymer and the fibre as a result of inadequate impregnation and wetting during the compounding process, and the third is the diffusion of water molecules via microscopic cracks in the polymer matrix as a result of fibre swelling [54,55,56,57]. These mechanisms allow the moisture diffusion in polymeric composites to be further classified as either Fickian or non-Fickian, which determines how quickly the material can absorb water [52,53,54,55,56,57,58,59]. Composite materials with high diffusion coefficients tend to absorb large quantities of water quickly, compared to those with low diffusion coefficients. Equation (1) is commonly used to determine the Fick coefficient.

(1) $$D=\pi {\left(\frac{kh}{4M_{m}}\right)}^{2\;}$$

where *D* is the Fick coefficient, *M_m_* is the maximum moisture content at equilibrium, *h* is the thickness of the composite, and *k* is the initial slope of the moisture content (*M(t)*) as a function of the square root of time (*t*^1/2^), as expressed by Equation (2).

(2) $${\left(\frac{M_{2}-M_{1}}{\sqrt{t_{2}\;}-\sqrt{t_{1}}}\right)}^{2\;}$$

In addition to Fickian diffusion, water absorption tests in the literature for NFBPC are commonly performed according to ASTM 570-98, whereby the percentage of moisture absorption (*M_c_*) by the composite is determined on the basis of Equation (3) [60,61,62,63,64,65,66,67,68]. In other cases, the percentage of thickness (*TS*) or the dimensional stability due to the absorption of water molecules is determined using Equation (4) [50,51].

(3) $$M_{c}=\left(\frac{M_{t}-M_{0}}{M_{0}}\right)\times 100\%$$

where *M_t_* is the weight of the sample at time *t* and *M*_0_ is the initial weight of the sample,

(4) $$TS=\left(\frac{T_{h}-T_{0}}{T_{0}}\right)\times 100\%$$

where *T*_0_ and *T_h_* are the thickness of the sample in dry and wet states, respectively, at a given time.

**Figure 3:**
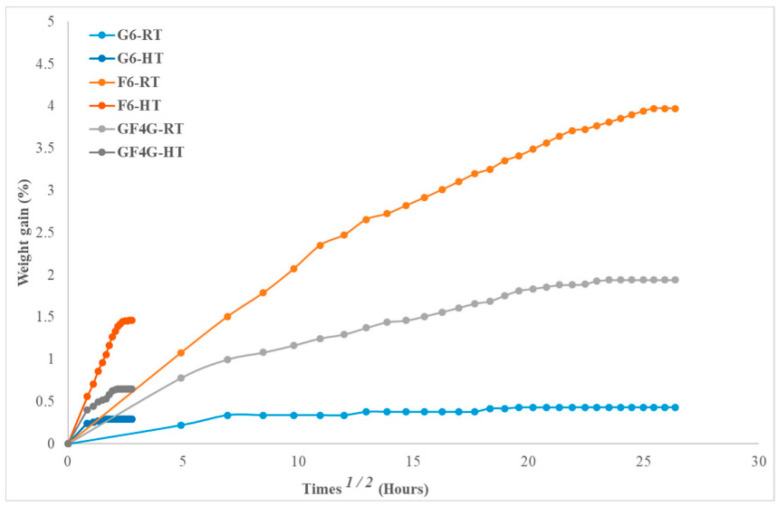
Moisture absorption versus square root of time at room temperature and high temperature [78].

Various studies in the literature in the form of reviews [65,66] and research papers [51,67,68,69,70,71,72,73,74,75,76] have described the use of chemical and physical treatments to overcome water absorption by natural-fibre-based composites. Another option for addressing some of the drawbacks of natural fibre composites is the hybridisation process, which enables the properties of the composites to be customised [77]. The use of hybrid composites has the benefit of producing materials at a reasonable cost while preserving their excellent thermal and mechanical properties. Moreover, hybrid composites afford balanced mechanical performance in comparison with single-fibre-reinforced composites. Hence, one of the factors that can determine the manner in which a composite material will absorb water is the hybridisation of the natural laminated fibre [77,78,79,80,81]. A previous study investigated the influence of moisture absorption on flax and flax/glass hybrid laminates with the aim of determining their low-velocity impact behaviour [78]. The highest percentages of moisture absorption for flaxfibre (F6)-, flax/glass hybrid (GF4G)-, and glass fibre (G6)-reinforced composites submerged at room temperature for 696 h were reported to be ~4%, ~2%, and ~0.4%, respectively (Figure 3). Furthermore, the composites’ moisture absorption behaviour under ambient conditions was shown to exhibit Fickian behaviour (Table 4). In the case of the flax fibre, high water absorption was expected due its having a high cellulose content (70%), causing a high rate of water intake. Water penetrates into the interface through the microscopic cracks, causing the fibres to inflate even more. As is well known, fibres possess several hydroxyl groups (-OH) in their structural makeup, and these groups form hydrogen bonds within the macromolecules of the cellulose. Hence, the presence of large numbers of –OH groups leads to high moisture absorption, thereby resulting in poor interfacial bonding between the polymer and the fibre. For the glass fibre samples, water penetrated through the matrix via voids created during the compounding process. However, the hydrophobic nature of the glass fibres limited the water uptake, similarly to the case of the hybrid composites. When compared to a flax/vinyl ester composite without hybridisation, the hybrid composite showed greater load and energy, indicating that the hybrid system was a viable approach for enhancing the structural performance of natural fibre composites.

Tezara et al. [77] examined the influence of the hybridisation of jute and ramie on the tensile and water absorption properties of epoxy resin. The authors investigated the moisture absorption behaviour of epoxy resin reinforced by jute and ramie hybrids. Additionally, the influence of stacking sequence hybridisation of the jute and ramie composite on water absorption was examined. Their results showed that after being submerged in water for 28 days, the specimen’s mechanical characteristics and water absorption behaviour, particularly its tensile strength, tensile modulus, weight increase, thickness swelling, and deterioration of tensile properties, were improved.

Therefore, one option to enhance the performance of jute fibre is to hybridise it with fibres that have greater mechanical qualities (such as ramie fibre), as jute has poor mechanical properties. This could yield superior results compared to processes in which the natural fibres are chemically modified. It was further concluded that employing the proper stacking sequence (R-J-R-J-R) is an essential factor in defining the quality of the hybrid composite in terms of attaining maximum mechanical strength.

On the other hand, at elevated temperatures, the moisture uptake of the resultant hybrid specimen does not exhibit Fickian diffusion behaviour, unlike at room temperature. This is due to the development of micro-cracks on the surface and inside of the hybrid as a result of the high temperature environment and the moisture [54]. Therefore, the material’s structural integrity is compromised, and cracks propagate in the form of resin particles, creating a more active water transport mechanism. The influence of temperature on water absorption behaviour was highlighted by Dhakal et al. [52]. The authors indicated that a larger moisture uptake occurred at higher temperatures. As a result of heat, the increase in temperature caused an increase in water absorption, which has a higher diffusion coefficient in high-temperature environments than at ambient temperature. Therefore, heat promotes thermal expansion, which weakens the contact between the fibre and the matrix. This leads to debonding and easy water molecule movement by providing an ideal pathway for water to move along at greater speed. This further causes fibre swelling, which results in the formation of matrix cracks and accelerates the deterioration of fibre–matrix interation. Improved reduction in water absorption by hybrid composites is highly dependent on the network structures that can be formed between the fibres and the polymer matrices.

## 5. Properties of Bast-Fibre-Based Hybrid Composites

Bast fibres can be combined with other fibres, e.g., synthetic or natural fibres, as well as fillers, to subsequently reinforce the polymeric matrix in order to develop hybrid composite. Hybridisation describes the use of optimal combinations of fibres and fillers to improve the properties of the resulting hybrid materials. This balances the advantages and disadvantages of the reinforcements, as well as their cost and performance, which can be determined using an appropriate experimental design. The properties of hybrid composites depend on various factors, including fibre dimensions, fibre loading, distribution of fibres in polymer matrix, and interfacial adhesion between fibres and polymers. The optimum results are attained when the fibres are extremely strain compatible [80].

### 5.1. Mechanical Perfomance of Bast-Fibre-Based Hybrid

**Figure 4:**
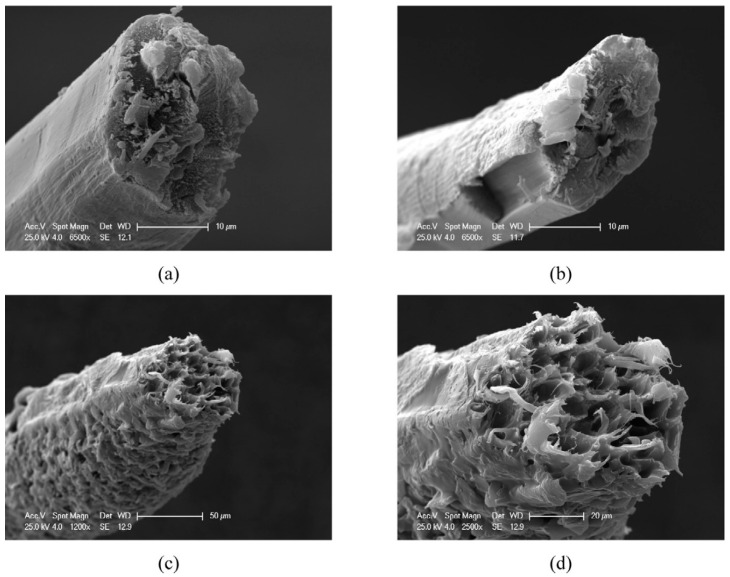
Scanning electron microscopy (SEM) images of ruptured tension fibres: (**a**,**b**) ramie and (**c**,**d**) borassus. Reprinted with permission from [44].

The incorporation of fibres into hybrid composites can positively and negatively influence the overall mechanical performance of the material or not influence it at all, especially when low loadings are incorporated. Prior to the fabrication of hybridised composite materials, the mechanical performance, density and morphological properties of each individual fibre and/or bulk fibre should be investigated in order to determine which fibres have greater levels of influence than the others. Natural fibres are isolated from different parts of the plants, i.e., leaf, stem, stalk, seeds, fruit, bast, etc., and offer different properties. This provides a platform from which to tailor the overall mechanical perfomance of the resulting hybridised materials. For instance, the mechanical properties of individual fibres (ramie and borassus) and bulk fibres were examined and compared by Sarasini et al. [44]. The results demonstrated that ramie fibres were brittle (as seen in Figure 4a,b), whereas borassus fibres were ductile, as depicted in Figure 4c,d. The ductility behaviour of borassus fibres was due to their low degree of crystallinity and high microfibrillar angle, as well as the fact that cellulose crystals were poorly oriented along the fibre axis. For bulk fibres, the surface of ramie fibres was smooth with defects. On the other hand, borassus fibres appeared to be rougher, irregular, and more compact, with no fibrillation. The results of the mechanical properties of the fibres demonstrated that the tensile strength and Young’s modulus of ramie fibres were superior to those of borassus fibres. In addition, ramie fibres displayed low strain to failure in comparison to borassus fibres. Similar studies have also been performed to investigate the mechanical properties of individual fibres prior to hybridisation [33,34,40]. From those studies, it was evident that bast fibres exhibit superior tensile strength and Young’s modulus compared to other natural fibres.

#### 5.1.1. The Effect of Fibre Treatment on the Properties of Bast-Fibre-Based Hybrids

**Figure 5:**
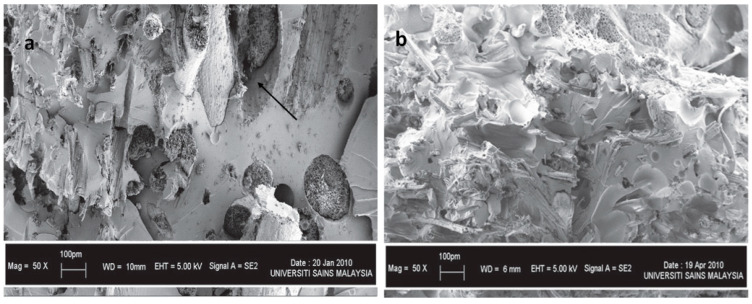
SEM images of tensile fractured composite materials made of (**a**) OPEFB:jute (4:1) and (**b**) OPEFB:jute (1:4). Reprinted with permission from [33].

As previously mentioned, bast fibres have been widely used in hybridisation processes to improve the overall performance of the resulting materials. Despite their usefulness in hybrid applications, there are some inherent drawbacks. These drawbacks include inherent polar and hydrophilic nature, low detectable defibrillation, which causes non-uniform dispersion within the polymer matrix, and a smooth surface, which leads to slippery fibre, resulting in fibre pullout and debonding, which can be attributed to the poor interfacial adhesion between fibres and polymers. This therefore leads to the resulting hybrid material possessing undesirable properties. For instance, Jawaid and coworkers [33] developed an epoxy hybrid composite reinforced with untreated jute and oil palm empty fruit bunch fibres (OPEFB). Fibre pullout and debonding could be observed in the SEM micrographs of hybrid composite materials that contained significant amounts of OPEFB, as depicted in Figure 5a (shown by arrow). Even though pullout and debonding were evident in the hybrid composite materials, it was noticed that the increase in jute fibre loading resulted in an improvement of the interaction between fibres and polymers, as shown in Figure 5b. The investigators suggested that it was because jute fibres have high tensile strength, allowing them to tolerate tensile stress. Moreover, the improvement of interfacial adhesion consequently led to better mechanical perfomance of the ensuing hybrid materials. Similar findings have been disclosed in other studies [81,82,83,84,85,86].

**Figure 6:**
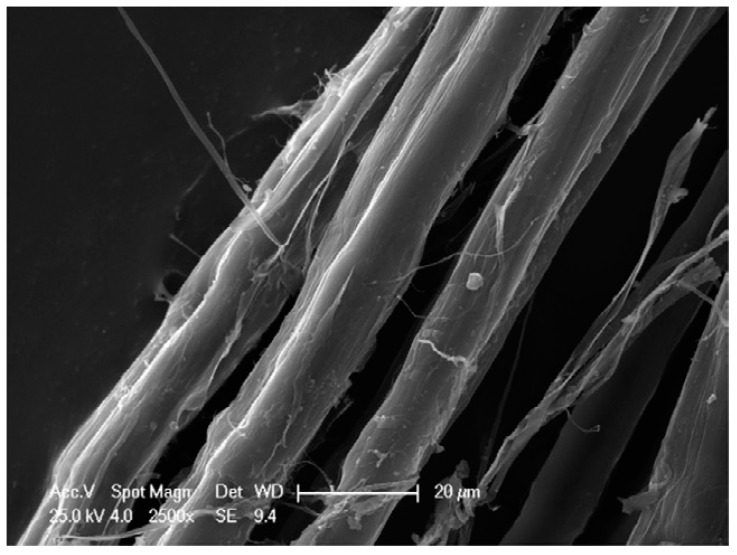
SEM images of alkaline-treated ramie fibres. Reprinted with permission from [44].

In order to mitigate the aforementioned shortcomings of bast fibres, one of the major strategies is the chemical treatment of fibres in order to enhance their dispersion within the polymer and improve the interaction between them. The treatment of bast fibres is usually achieved by silane, alkaline, grafting, and acetylation. Alkali treatment using sodium hydroxide has been widely employed for treating bast fibres. The treated fibres were subsequently reinforced with hybrid composites with the aim of improving their interfacial adhesion and mechanical properties. Numerous researchers [44,83,84,85,86,87] have studied the effect of the chemical modification of bast fibres on the mechanical performance of the resulting hybrid composite samples. The results have demonstrated that the chemical treatment of fibres leads to strong interaction between fibres and polymers, thus enhancing their performance. Elsewhere, 5 wt.% NaOH-treated ramie fibres were incorporated into composite samples to afford the production of bast hybrid composite materials [44]. The alkali treatment afforded the defibrillation of fibres into individual fibres due to the removal of cementing hemicellulose component and other extractives, thus reducing their diameters, as shown in Figure 6, which improved their aspect (length-to-diameter) ratio.

This increased their surface area, thus allowing the polymer to penetrate between the individual fibres. Therefore, this resulted in a strong interaction between the fibres and the polymeric matrix, leading to an enhancement of the overall mechanical performance of the hybrid. The incorporation of the alkaline-treated ramie fibres into the polycaprolactone (PCL) hybrid composite containing borassus fibres resulted in a notable increase in both tensile strength and Young’s modulus, whereas ductility behaviour decreased. In fact, this is in agreement with the hypothesis that the tensile properties of composites improve when reinforced with fibres possessing a high aspect ratio. This increment is caused by good stress transfer from the polymeric matrix to the fibres, and the fibres are able to withstand stress. It has also been reported that the incorporation of treated ramie fibres does not influence the hardness of the ensuing composite samples. Other researchers have investigated the impact of treating kenaf and pineapple fibres with silane on the tensile characteristics of epoxy hybrid composite materials [87]. The silane treatment positively influenced both the tensile and flexural properties, because the treatment promoted strong interaction between the fibres and the polymeric matrix. The interaction between the materials was also confirmed by Fourier-transform infrared spectroscopy (FTIR). The results for tensile properties correlated with the morphological results, as the hybrid composites did not display debonding and fibre pullout. Additionally, the surface treatment of the fibres increased the composites’ impact properties. On the other hand, the hardness values of the hybrid reinforced with treated fibres were lower than those of the hybrid reinforced with untreated fibres.

**Figure 7:**
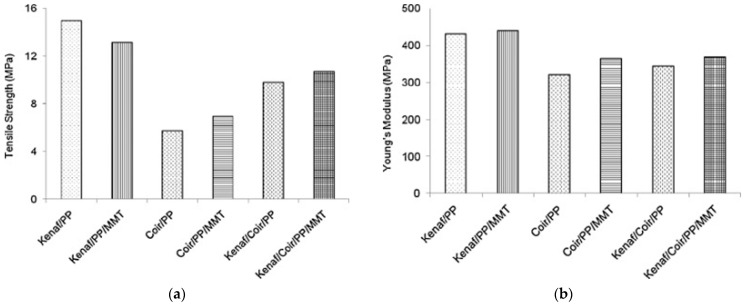
(**a**) Tensile strength and (**b**) Young’s modulus of PP-based composites, *viz*., kenaf-PP, coir-PP, kenaf-coir-PP and their nanocomposites. Reprinted with permission from [88].

Islam et al. [88] reported that the incorporation of treated kenaf and coir with 2 wt.% NaOH into polypropylene (PP) slightly improved the interaction between fibres and the polymeric matrix. FTIR confirmed that the alkali treatment reduced the hydroxyl and carbonyl functional groups, thereby making the fibre more reactive, leading to good interaction with the polymer. The incorporation of the treated fibres into PP resulted in physical interaction between the polymer and the fibres. Upon introduction of montmorillonite (MMT) into hybrid composites, a significant improvement was evident due to enhancement of interfacial bonding between the fibres and the polymeric matrix. Thus, fibre debonding and pullout were significantly decreased. These findings also reflect that the incorporation of MMT results in a significant improvement in the tensile properties of the composites, as shown in Figure 7 [88].

#### 5.1.2. Effect of Loading of Bast Fibres on the Tensile Properties of Hybrid Composites

The incorporation of fibres has been reported to improve the tensile properties of the resulting hybrid materials [33,44,89,90]. Even though the incorporation of bast fibres has been demonstrated to enhance the tensile properties, nevertheless, numerous researchers have reported that increasing fibre loading results in the enhancement of tensile properties [33,44,89,90,91,92,93]. For example, Jawaid et al. [33] investigated the influence of natural fibres (OPEFB and jute) at various loadings on the tensile properties of the ensued hybrid materials. The results demonstrated that the introduction of 20 wt.% jute fibres into epoxy hybrids reinforced with OPEFB led to an improvement in the tensile properties, as depicted in Table 5.

However, a significant improvement was noticeable when jute loading was increased from 20 to 80 wt.%. Similar findings have been reported in other studies [33,44,90,91,92,93]. In these studies, it was reported that when the bast fibre loading was increased, the mechanical properties also increased, whereas tensile strain decreased. The improvement in tensile properties was due to the high tensile strength and modulus of the bast fibres. With increasing loading, bast fibres are able to withstand higher loads, while distributing lesser loads to other fibres in a hybrid material leads to enhanced tensile properties [33]. In addition, the incorporation of bast fibres increases the load-bearing capacity, which results in improved stiffness.

### 5.2. Viscoelastic Properties of Bast-Fibre-Based Hybrid

Dynamic Mechanical Analysis (DMA) is the most effective technique for determining the viscoelastic properties (stiffness and damping behaviour) of polymeric materials such as hybrid materials [94]. DMA monitors the manner in which viscoelastic behaviour varies with temperature, time, and frequency. The stiffness of the material is determined using storage modulus, whereas Tan δ is used to determine the elastic behaviour, viscosity, and impact properties of the polymeric material. The glass transition temperature (T_g_), which is attained on the basis of damping behaviour, defines the temperature range in which the polymeric material transforms from a glassy to a rubbery state [93]. Comparably to mechanical properties, DMA depends on various factors, such as the fibre treatment, the orientation of the fibre, the fibre type, the fibre loading, etc. It is evident from previous studies that DMA provides valuable information about the changes in the viscoelastic behaviour of hybrid materials [42,91,92,93,94,95,96,97].

In an intriguing work by Pappu et al. [42], the viscoelastic behaviour of a PLA hybrid reinforced with sisal-hemp fibres was studied. The authors reported that the storage and loss modulus of the hybrid samples were higher compared to virgin PLA. The storage modulus of all investigated samples decreased with increasing temperature indicating that these materials were transforming from a glassy to a rubbery state. The area under the Tan δ curve decreased in hybrid composites, indicating an increase in the damping ability of hybrid composites when compared to PLA. T_g_ was unaffected by the fibre’s addition to PLA. Jawaid et al. [97] obtained similar findings. In their study, they fabricated epoxy hybrids reinforced with OPEFB and woven jute fibre. In all investigations, the presence of the bast fibres in the hybrid material was credited with enhancing the viscoelastic properties. A similar pattern was observed for hybrid composites reinforced with OPEFB nanofillers [94].

**Figure 8:**
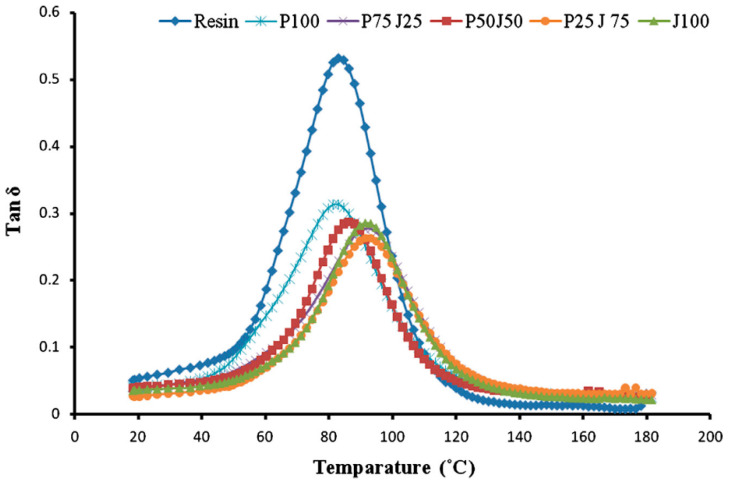
The variation of Tan δ with temperature for an unsaturated polyester hybrid reinforced with palmyra palm and jute fibres [91].

Other researchers [42,91,96] have investigated the effect of fibre loading on the viscoelastic properties of hybrid samples. In general, because of the increase in fibre loading, the viscoelastic properties are improved. For instance, Shanmugam and Thiruchitrambalam [91] investigated the viscoelastic behaviour of an unsaturated polyester hybrid reinforced with palmyra palm and jute fibres with various fibre loadings (75/25, 50/50, 25/75). It was observed that the storage modulus of the hybrid composites gradually decreased when compared to the virgin resin. This behaviour was due to the presence of bast fibres with high tensile modulus. It was noted that the increase in fibre loading increased the storage modulus, and the hybrid with a high jute fibre loading had a high storage modulus. The Tan δ of hybrid composites was reduced due to the reinforcement effect of fibres, thus indicating good interfacial adhesion between the composite’s components. A good interfacial adhesion between the components of the composite reduces the chain mobility of polymers, thereby reducing the damping factor. It is worth noting that the higher the jute fibre loading, the lower the peak height compared to other composites. Moreover, the T_g_ of the composite shifted to higher temperatures, as depicted in Figure 8 [91].

In another study, the effects of frequency on the viscoelastic behaviour of epoxy hybrid materials reinforced with sisal and jute fibres were investigated [95]. Hybrids demonstrated improved storage modulus, loss modulus, and T_g_ compared to neat epoxy and epoxy reinforced with sisal fibres alone. This improvement became apparent with increased jute fibre loading. Additionally, the improvement of the viscoelastic properties of the hybrid composite materials became evident when the frequency was increased from 1 Hz to 10 Hz.

## 6. Thermal Properties of Bast-Fibre-Based Hybrid

In-depth thermal properties analyses of bast-fibre-based hybrids have been performed previously [42,98,99,100,101,102]. These studies focused on the effect of fibre treatment as well as varied fibre loading. Thermal decomposition and stability, as well as melting behaviour, were measured to determine the thermal properties of the hybrids. Thermogravimetric analysis (TGA) is used to study thermal decomposition behaviour and stabilities of materials, whereas differential scanning calorimetry (DSC) is used to investigate their melting behaviour. It has been reported that the incorporation of fibres does not change the degradation pattern of the hybrid composites [42,98,99]. However, the introduction of bast fibres into hybrid materials improves their thermal stability due to the bonding of the fibres with the polymeric matrix and the high thermal stability of the bast fibres [97]. The thermal stability of hybrid materials is dependent on the properties of both fibres: the reinforcement and the polymeric matrix. For instance, Cavalcanti et al. [98] studied the thermal stability of composites as a function of hybridisation and polymeric matrix type. The hybrid composites investigated included jute + curauá-based epoxy, jute + sisal-based epoxy, jute + curauá-based polyester and jute + sisal-based polyester. The results revealed that non-hybrid jute-based epoxy had higher onset temperatures in comparison to the other hybrids. This was due to the high loadings of highly hydrophilic fibre with a tendency towards moisture absorption, thereby reducing thermal stability. It is worth noting that jute + sisal-based epoxy was more thermally stable, followed by jute + curauá-based epoxy and then non-hybrid jute-based epoxy. The opposite pattern was witnessed for polyester-based hybrids, due to the lack of bonding between fibres and polymers and the lack of chain mobility. Another study revealed that increasing the ramie fibre loading in an epoxy hybrid improved the materials’ thermal stability [27]. Conversely, in the case of epoxy hybrid reinforced with both jute and glass fibres, increased jute fibre loading with decreased glass fibre loading resulted in a decrease in the thermal stability of the resultant material [100].

The effect of fibre treatment on the thermal stability of hybrid composites has also been investigated [98,100,101]. It is well known that the chemical treatment of fibres with alkaline removes impurities and components that are thermally less stable. However, these researchers observed that the decomposition temperature of hybrid reinforced with treated fibres shifted to a higher temperature, indicating an improvement in thermal stability.

Few studies have investigated the melting and crystallisation behaviour of hybrid composites [42,99,101,102]. The melting temperature (T_m_), melting enthalpy (Hm), crystallisation enthalpy (H_c_), crystallisation temperature (T_c_), and degree of crystallinity (X_c_) were determined via analysis in order to fully understand the thermal properties. In these aforementioned studies, it was reported that the incorporation of bast fibres into hybrids influences their melting and crystallisation behaviour. For instance, Aisyah et al. [99] reported that the inclusion of woven kenaf in an epoxy hybrid reinforced with carbon fibre negatively affected the T_m_ and T_c_ of the hybrid. This was caused by the inclusion of weaved kenaf, which reduced the amount of total energy needed to be absorbed to break polymer chains. Sarasini et al. [44] indicated that the introduction of ramie and orassus fibres into PCL improved crystallinity, T_m_ and T_c_, while the crystallisation enthalpy (∆H_c_) and melting enthalpy (∆H_m_) had lower values. However, it is worth mentioning that there were no significant changes in crystallinity, T_m_ or T_c_ when fibre loading further was increased from 10 to 30 wt.%. The influence of chemical treatment on the thermal behaviour of hybrid materials has also been studied [101]. The incorporation of treated fibres with alkaline into hybrid composites led to an increase in T_g_ when compared to hybrid reinforced with untreated fibres, while the endothermic peak decreased in hybrids reinforced with both untreated and treated fibres.

## 7. Flame Resistance

Most hybrid composite applications require them to meet certain fire regulatory criteria in order to ensure public safety [5,103,104,105,106]. To examine the combustion performance of different hybrid products, a wide variety of techniques can be employed, depending on the desired application. Cone calorimetry is often used for simulation of real-life situations [4,106,107]. It provides useful data related to combustion of materials, such as heat release rate (HRR), total smoke release (TSR), carbon dioxide production (CO_2_P), total heat release (THR), and smoke production rate (SPR). For instance, pHRR provides essential data based on the intensity of the fire, *viz.,* higher pHRR values indicate a major fire hazard. Owing to the high flammability of polymeric materials and natural fibres, different additional fillers can be incorporated to improve their flame-retardant properties [4,5,103,104,105,106,107,108,109]. Table 6 summarises the results obtained for bast-fibre-based hybrid composites.

**Figure 9:**
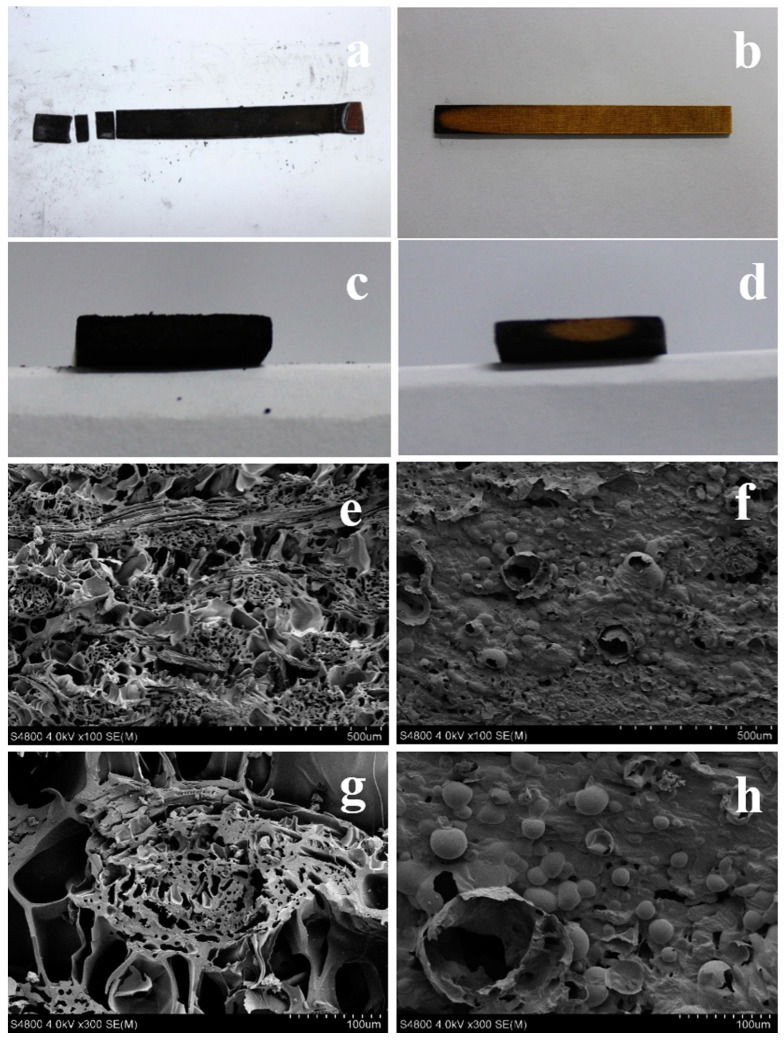
Images of the char residues for (**a**,**c**,**e**,**g**) untreated ramie hybrid composites and (**b**,**d**,**f**,**h**) treated ramie hybrid composites. Reproduced from [24].

Silane treatment of bast fibres in flame-retardant hybrid systems has been reported in the literature [25,26]. A comparison between the treatment of silane-treated fibres with APP (AF) and introducing and mixing APP into PLA (APLA) before the introduction of silane-treated fibres was conducted by Li et al. [24]. It was reported that the incorporation of APP into PLA is an acceptable method for producing hybrids with acceptable flame-retardant properties. The composites exhibited LOI values of 28–37, with UL94 V0 rating. Li et al. [24] treated ramies fibres with silane followed by decoration with ammonium polyphosphate (APP), and polyethylenimine (PEI) to afford flame retarded hybrid composite materials. The resultant composite exhibited excellent flame retardancy reaching LOI value of ~39%, with UL-94 V-0 rating. The untreated composites were flammable, leading to brittle fracture and shrinkage after fire testing (Figure 9a,c). The treated ramie-based composites easily quenched the flame after being removed from the burner with (Figure 9b,d). Through the evaluation of the collected char after fire testing, it was deduced that treatment improved the flame resistance performance, as indicated by the formation of bubbles on the surface of the specimen. This was ascribed to the presence of APP and PEI facilitating the formation of foam from their combustion products (e.g., phosphoric acid, ammonia, water, metaphosphoric) that acted as a protective layer. The untreated specimens exhibited a loosely delaminated structure with numerous holes, indicating acute decomposition.

**Figure 10:**
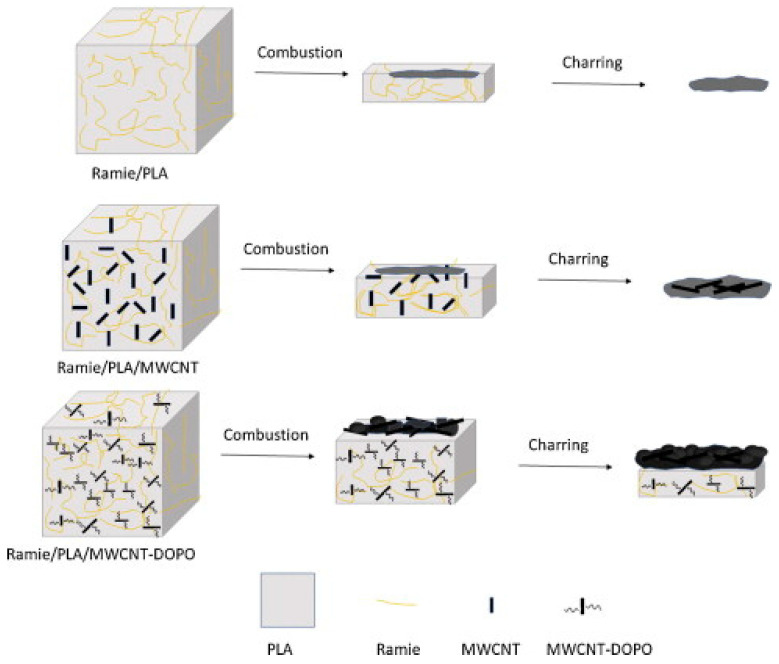
Schematic representation of flame resistance mechanism of ramie composites and their hybrids. Reproduced from [110].

Modification of the secondary filler rather than the bast fibres can also be adopted in order to improve the overall flame-retardant property of the resulting hybrid composites [110,111]. The treatment of the CNTs with a mixture of acid to introduce carboxyl groups afforded chlorination with thionyl chloride, resulting in functionalisation with 10-dihydro-9-oxa-10-phosphaphenanthrene-10-oxide (DOPO) [110]. The resultant hybrid composite composed of ramie/CNT-DOPO/PLA had higher char residue than the untreated CNTs/ramie/PLA. This was a result of the ramie fibres acting as a carbon source for charring, in turn acting as a shield to protect the underlying materials. This layer delays the transportation of volatiles and heat transfer, thus promoting flame resistance performance, as shown in Figure 10.

In situ synthesis of phosphorus- and nitrogen-containing FR on the surface of ramie fibres was carried using condensation reaction by Du et al. [103]. The hybrid was prepared using melt blending in the presence of PP-grafted maleic anhydride (PP-g-MAH) as a compatibiliser. pHHR decreased from 714 kWm^−2^ (PP/RF) to 547 kWm^−2^ (PP/RF/FR), and THR decreased from 96 MJm^−2^ to 84 MJm^−2^, respectively. It was concluded that the FR on the surface of the fibres degraded, creating a continuous char residue that protected the fibres from the heat source. Elsewhere, both melamine pyrophosphate (MPP) and aluminium hypophosphite (AP) were introduced into BF/PP composites at a mass ratio of 2:1 [108]. The addition of 30% FR resulted in pHRR and THR reduction by ~39% and ~21%.

Metallic hydroxides are known for their flame-retardant properties, which result from a cooling effect due to endothermic reactions from their degradation [26]. The incorporation of metallic hydroxide into hybrid composites was also studied [26]. In their study, El-sabbagh et al. [26] studied the influence of magnesium hydroxide (Mg(OH)_2_) on the flame retardance of PP/flax composite materials with PP-g-MAH employed as a compatibilising agent. It was pointed out that despite the samples failing to satisfy the lowest UL rating, the samples exhibited a prolonged burning process without signs of dripping. This was ascribed to low content of Mg(OH)_2_ (i.e., 30%), since 50–60% is required to achieve the desired flame-retardant properties. In addition, a recyclability study on this composite indicated that an increase in the number of recycling cycles led to a reduction in flame resistance performance by almost 5%. The preparation method plays a critical role in the dispersion of the fillers, and thus the resultant properties. Zhang et al. [112] prepared hybrid composites made up of PP:hemp fibres: APP: organomodified montmorillonite:MA-PP 35:35:30:5:5 using three different methods of mixing the ingredients. The first process involved the ingredients being added into a melt mixer and blended; in the second, the ingredients were divided into three batches and added to the mixer and blended three times; and the third involved hemp being immersed in APP solution, dried, and then mixed with other ingredients. Despite all composites having the same LOI value of 29%, the second method afforded thermally stable specimens because of the OMMT being closely distributed on the surface of the hemp fibres, and thus protecting them from heat and mass transfer. In the case of the first method, the OMMT particles were evenly distributed throughout the system, rather than on the surface of the hemp fibres, and thus failed to protect the underlying material. In the third processing method, the hemp fibres were decorated with APP, while the OMMT were distributed within PP, leading to poor thermal properties. It is worth noting that this composite performed better with respect to mechanical and viscoelastic properties. This demonstrates that the processing method can be chosen based on the intended application [112].

The incorporation of FR into bast-based composites can drastically improve the flame resistance performance of the resulting hybrid composite materials [112]. The use of these FRs as modifiers for the fibres serves as a suitable alternative for improving adhesion between the polymers and fibres, while maintaining the desired flame resistance performance. The optimisation of the contents of the components and the processing method are critical for attaining acceptable flame-retardant properties.

## 8. Applications of Bast Hybrid Composites

Synthetic fibres play a critical role in the resulting properties of bast hybrid composites [12,20]. The enhanced stability with reduced moisture adsorption opens doors for their application in different fields. In addition, the treatment of bast fibres can reduce their moisture absorption and enhance their interaction with hydrophobic polymers, thus widening their application. The inclusion of flame retardants gives bast-fibre-based hybrid composites additional functionalities for applications where fire safety is of the essence, e.g., in automotive and aerospace applications, etc.

### 8.1. Automotive

**Figure 11:**
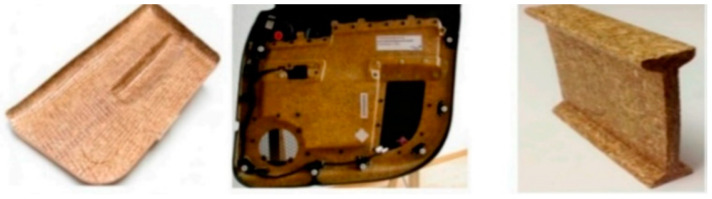
Automotive parts developed from natural-fibre-based composites. Reproduced from [113].

**Figure 12:**
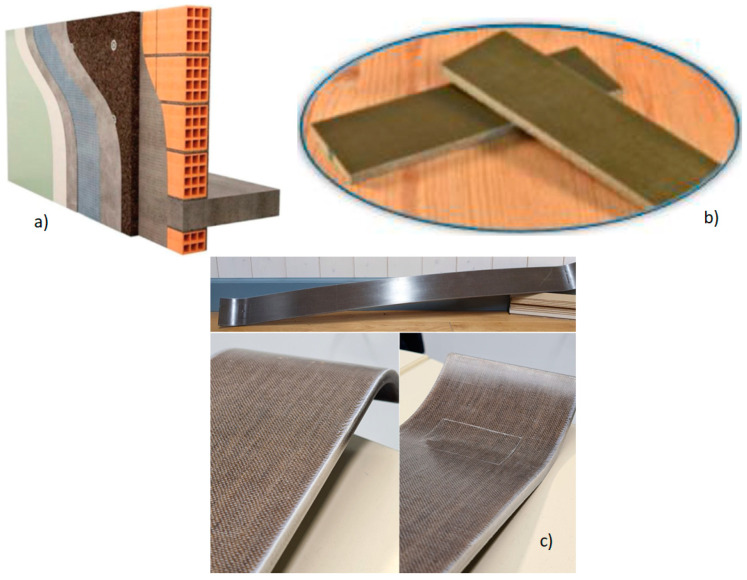
Natural-fibre-based composites for various applications: (**a**) insulation. reproduced from [114]; (**b**) panel, reproduced from [115]; and (**c**) leaf spring of a bogie of a narrow-gauge railway, reproduced from [116].

On the other hand, the light weight of bast fibres has been explored for automotive applications, such as door panels, armrests, seat backs, etc., as depicted in Figure 11 [113]. Other applications of bast-fibre-based composites include panels, insulators, and railways, as depicted in Figure 12a–c [113,114,115,116,117]. The main aim of incorporating bast fibres is to reduce the weight of the car, thus improving fuel efficiency [12]. In addition, these fibres reduce the overall cost of these components, since they are abundantly available at fairly low cost. PLA hybrid composites have been reported to be applicable for the production of automotive parts, electronics, interiors, and agricultural products [40]. This is as result of their biodegradability, and their excellent mechanical properties with low water absorption. Due to their light weight, bast hybrid composites can be used to replace wood products. In addition, the mechanical strength of these composite materials is superior compared to classic wood. The resulting products exhibit superior properties when compared to wood, including: good dimensional stability, excellent weather resistance, remarkable impact resistance, and acceptable flame resistance performance, while being cheaper due to low maintenance costs.

### 8.2. Biomedical Applications

The use of biodegradable polymers (e.g., PCL) opens doors for the application of hybrid composites in biomedical applications [44]. Sarasini et al. [44] prepared hybrid composites from PCL reinforced with ramie and borassus fibres. The resulting mechanical performance was similar to commercially available materials for orthotics, indicating that the as-prepared hybrid could be used for manufacturing orthotic devices due to the synergy between the reinforcing fibres.

### 8.3. Aerospace

The unique features of natural fibres, such as their light weight, inexpensiveness, and sustainability, have led them to be explored as alternative fillers for composite materials to be used in aircraft components [117]. Aircraft radomes are radar-transparent dome-shaped structures that shield radar antennas against aerodynamic loading, weather, and impacts when struck by objects, and they can be covered with bast-fibre-based hybrid composites. Haris et al. [117] indicated that kenaf is one of the suitable reinforcements for various polymers used in the fabrication of aircraft radomes. The materials were chosen based on valuable properties, starting from dielectric to mechanical performance. It was also indicated that it is necessary for the material to withstand harsh weather conditions and possess excellent impact properties. Therefore, the combination of kenaf with other fillers can afford the production of hybrid materials with acceptable properties for producing covers for aircraft radomes. In addition to the high annual yield of bast fibres, their noise absorption and mechanical performance can be explored for manufacturing the internal components of aircraft [118].

### 8.4. Other Applications

The moisture absorption of bast-fibre-reinforced composites is often a limiting factor in water-related applications. The moisture absorption of bast-fibre-reinforced composites can be solved through hybridisation with synthetic fibres, such as glass or carbon fibres. Fiore et al. [119] demonstrated that the hybridisation of jute with basalt fibres improved the durability of the hybrid structures under salt fog aging conditions. This demonstrated that these composites could be used in marine applications. In addition, the hybrid composites showed ~98% and ~90% recovery of their initial stiffness and strength after 30 days of salt-fog spay treatment [120].

## 9. Outlook

Great strides have been made in recent years with regard to the preparation and characterisation of bast hybrid composites. This is a result of strict regulations necessitating the design of products that are eco-friendly and sustainable. In addition, the resulting properties are comparable to those of conventional synthetic hybrid composites; thus, they can be used in a variety of applications (e.g., automotive, furniture, construction, biomedical, etc.). Bast fibres can be exploited as suitable natural fibres to reduce the amount of synthetic fibres often incorporated into traditional synthetic-based hybrid composites. Their combination with synthetic fibres lowers the total cost of the final product and improves the overall performance of the resulting composite materials. Despite the fact that the use of other natural fibres as secondary fillers in bast hybrid composites has been reported to improve the overall resulting properties, the modification of these fibres is essential to promote adhesion while decreasing the moisture absorption of the resulting products. In future, some of the modifications that can serve as flame retardants while improving hydrophobicity will revolutionise the use of natural fibres as reinforcing agents in composite products. Synthetic flame retardants are commonly employed as fillers to improve the flame resistance performance of bast-based hybrid composite products. In future, the use of natural-based FRs should be explored for the development of highly flame-retardant eco-friendly materials.

This review’s main focus was on the production of bast hybrid composites using various processing techniques. It is worth mentioning that short or long fibres can be employed, depending on the host matrix and the processing technique used. Treatment of the fibres prior to composite preparation is often performed to increase fibre roughness to facilitate interfacial interlocking with the polymeric materials. Alkali treatment is a commonly used treatment for bast fibres. The resulting properties of the hybrid composite products is directly influenced by the preparation method, the fibre treatment, the fibre dimensions, the fibre type, the alignment of the fibres within the system, and the content of the fibres. We also focused on the use of FRs as a secondary filler to improve the flame-retardant properties of the resulting bast hybrid composites. It was deduced that these FRs can be used as a form of treatment for the fibres, and thus optimisation is often required to achieve the desired flame-retardant properties. In the case of applications, the bast hybrid composites have a huge potential to replace traditional materials (e.g., wood) in some applications. Despite other properties (e.g., thermal and flame-retardant properties) being important for widening the application of hybrid composites, the most frequently investigated feature is the mechanical properties, followed by moisture absorption. This effect is due to the potential for using natural fibres in some applications rather than the commonly used synthetic carbon and glass fibres. There are very few studies centred on employing these methods to explore the impact of hybridisation, despite the rich information that other analytical techniques such as XRD, and FTIR may give with relation to the desired application. The majority of studies employ these techniques when bast fibre and/or polymer modification has been performed to increase interfacial adhesion between composite components. Therefore, more studies on other properties are still required to understand the overall factors influencing the resulting hybrid bast-fibre-based composites. The use of biological treatment is often limited to the removal of other constituent fibres. Studies based on the hybridisation of such fibres are also needed in order to understand the influence of such treatments on the properties of the resultant hybrid composites.

## 10. Conclusions

This review covered the preparation and characterisation of bast-fibre-based hybrid composites. It can be concluded that bast hybrid composites can be utilised in different applications, opening new platforms for the development of novel products that can compete with traditional hybrid composites. Different polymeric materials and processing techniques have been used to manufacture bast hybrid composites with attractive properties, such as renewability, low density, and cost effectiveness. In most cases, the fibres were chemical modified in order to improve their adhesion with the host polymeric materials. With various developing countries producing the majority of bast fibres, their use serves as a suitable alternative for achieving socio-economic empowerment. Studies using techniques other than traditional mechanical, thermal and moisture absorption are required to obtain insights on the influence of the hybridisation of bast fibres.

## Figures and Tables

**Table 1:** Bast fibre export and import by country 2021 Source: https://wits.worldbank.org/trade/comtrade/en/country/ALL/year/2021/tradeflow/Exports/partner/WLD/product/530310, accessed on the 9 September 2022.

Exporters	Trade Value in Millions of USD
India	31.5
Tanzania	14.5
Belgium	2.35
Netherlands	0.91
Malaysia	0.59
Unites States	0.57
Domican republic	0.43
European union	0.24
United Arab Emirates	0.16
Spain	0.11

**Table 2:** Properties of bast and synthetic.

	Bamboo	Flax	Hemp	Jute	Kenaf	E-Glass Fibre	Carbon	Kevlar	Basalt
Cellulose (%)	55–90	60–79	65–80	59–71	45–58	-	-	-	-
Hemicellulose (%)	4–16	16–18		12–13		-	-	-	-
Lignin (%)	2–5	3.0–4.5	3.0–5.0	11.8–12.9	12.0–13.0	-	-	-	-
Wax (%)		1.5	0.7	0.5		-	-	-	-
Pectin (%)	0.8–8	1.8–2	0.8	0.2–4.4	3.0–5.0	-	-	-	-
Angle microfibril (°)	2–12	5–10	2–6.2	8.1	9–15	-	-	-	-
Density (g cm^−3^)	1.52	1.3–1.5	1.52	13–1.45	1.45	2.5	1.7	1.3–1.5	2.7–2.8
Tensile modulus (GPa)	15.4–27.5	28–80	70	13–60	14–38	70	230–240	70–160	84–87
Tensile strength (MPa)	270–889	345–1500	690–920	393–860	240–930	2000–3500	4000	1240–4100	4000–4700
Elongation at break (%)	-	2.7–3.3	0.6–1.7	0.6–2.0	1.2–1.6	2.5	1.4–1.8	1.5–3.6	3.1–3.6
Cost (euro/kg)	-	1.3–1.4	5–10	1.2–1.6	1–3	0.46–2.56	26–34	-	0.34–3.42
Refs.	[3,12,18,19,20,21,22]	[12,19,20,22]	[12,19,22]	[12,19,21]	[12,19,22]	[12,19,21]	[12,19,22]	[12,19,20,22]	[12,19,21,22]

**Table 3:** Summary of studies on the preparation of bast hybrid composites.

Polymer	Hybrid Fillers	Fibre Treatment	Processing Technique	Content of Fibres (wt.%)	Intended Application	Refs.
PLA	Jute/hemp	Alkali treatment 5% NaOH at 30 °C for 3 h	Compression moulding	50	-	[41]
	Sisal/hemp	-	Extrusion and injection moulding	30	Automotive, packaging, electronics, interiors and agricultural applications.	[43]
PCL	Ramie/borassus	Alkali treatment (5% for 4 h at 25 °C)	Melt mixing	10–30	Orthotic devices	[45]
PP	Kenaf/pineapple leaf	Alkali (5% NaOH for 4 h) followed by silane (3% for 3 h) treatment	Melt mixing	25–50	-	[37]
Polyethyelene	Kenaf/snail shells	-	Compression moulding	9–24		[42]
Epoxy	Jute/palm	-	Hand-layup-compression moulding	40	Automotive and aerospace	[46]
Epoxy	Flax/carbon/basalt	-	Hand layup-compression moulding	-	Medium structural loading	[47]
Epoxy	Hemp/basalt/glass	-	Vacuum infusion	20.16	-	[50]
	Flax/basalt/glass			21.18		
	Hemp/basalt/glass			22.53		
	Flax/hemp/basalt			21.18		
Epoxy	Flax/Kevlar	-	Hand layup	32.5	Automotive	[48]
Unsaturated Polyester	Jute/newspaper waste	-	Hand layup-compression moulding	42%	-	[38]
Vinyl ester	Flax/basalt	-	Resin infusion and hand layup	-	Light-to-medium structural loading	[32]
Vinyl ester	Flax/basalt	-	Resin infusion and hand layup	-	Higher structural loadings	[32]
Vinyl ester	Flax/Carbon	-	Resin infusion and hand layup	-		
Phenolic	Jute/bagasse	Alkali + Oxidation + Furfural grafting	Hand layup and compression	30	-	[3]

**Table 4:** Moisture absorption and diffusion coefficient reported in various studies.

Samples	Moisture Absorption/%	Diffusion Coefficient/m^2^/s	Reference
G6	0.43	2.5 × 10^−9^	[78]
F6	3.97	1.4 × 10^−8^	
GF4G	1.94	8.5 × 10^−9^	
GM	0.41	1.51 × 10^−6^	[57]
SGH	4.99	6.64 × 10^−6^	
B7	17.9	1.2 × 10^−5^	[79]
S7	9.5	1.5 × 10^−5^	
BS7	14.4	1.6 × 10^−5^	
25	3.6	9.41 × 10^−2^	[59]
35	4.1	1.12 × 10^−2^	
45	4.3	1.15 × 10^−2^	

**Table 5:** Tensile properties of OPEFB:jute fibre-based hybrids [33].

Material	Tensile Strength (MPa)	Young’s Modulus (GPa)
Epoxy-OPEFB	~23	~2.2
Epoxy-(OPEFB:jute) (4:1)	~25	~2.6
Epoxy-(OPEFB:jute) (1:1)	~28	~2.9
Epoxy-(OPEFB:jute) (1:4)	~38	~3.3
Epoxy-jute	~46	~3.9

**Table 6:** Summary of flame-retardant properties of bast hybrid composites.

Hybrid System	Comments	Refs.
PP/ramie phosphorus oxychloride and further 4,4′-diaminodiphenylmethane (DDM)	pHHR and THR reduced by ~23% and ~13% when compared to composite material due to formation of continuous compact char residue on the fibres’ surface that shielded them from fire attack	[103]
PP/kenaf/water glass	Water glass treatment resulted in improved flame-retardant properties of the composites	[109]

## Data Availability

The data that support the findings of this study are available on request from the corresponding author.
